# Molecular epidemiology of *Nakaseomyces glabrata* associated with vulvovaginal candidiasis revealed high genetic variability and the presence of novel genotypes in China

**DOI:** 10.1080/21505594.2025.2543058

**Published:** 2025-08-03

**Authors:** Xue Xu, Yanxia Sun, Danyang Hu, Clement Kin-Ming Tsui, Lu Zhang, Shuwen Deng

**Affiliations:** a Department of Medical Microbiology, The People’s Hospital of Suzhou New District, Suzhou, China; b Life Science College, University of Essex, Colchester, UK; c Infectious Diseases Research Laboratory, National Centre for Infectious Diseases, Tan Tock Seng Hospital, Singapore; d Lee Kong Ching School of Medicine, Nanyang Technological University, Singapore; e Division of Infectious Diseases, Faculty of Medicine, University of British Columbia, Vancouver, Canada

**Keywords:** Molecular epidemiology, *Nakaseomyces glabrata*, vulvovaginal candidiasis, genotypes, China

## Abstract

*Nakaseomyces glabrata* (former name: *Candida glabrata*) is the second most common cause of vulvovaginal candidiasis (VVC), but its molecular epidemiology and antifungal resistance in China remain poorly understood. This study analysed 204 *N. glabrata* isolates from VVC patients in Suzhou, Eastern China, using multi-locus sequence typing (MLST) and microsatellite genotyping, alongside antifungal susceptibility testing. A total of 46 sequence types (STs) were identified by MLST, as well as 146 genotypes (GTs) revealed by microsatellite. According to MLST, ST7 was the predominant ST in vaginal *N. glabrata* isolates, along with a considerably high proportion (32/46, 69.6%) of novel STs. Notably, 27 STs were unique singletons, of which 25 unique STs were newly defined in this investigation. Microsatellite genotyping revealed a similar pattern as MLST with high variability. Population genetic analysis revealed evidence of recombination and ST7 could be the founding population of other related STs. However, there was no significant association between the genotypes and resistance phenotypes. Molecular epidemiology of *N. glabrata* associated with VVC revealed high genetic variability and the presence of novel genotypes in China. This study highlights the unique genetic profile of vaginal *N. glabrata* isolates in Suzhou, with the majority of resistant strains belonging to ST7.

## Introduction

Vulvovaginal candidiasis (VVC) is defined as a symptom of inflammation and excessive overgrowth of yeast, especially *Candida* species [1] in the vagina and the vulva. It is estimated that about 75% of women have been infected at least once in their lifetime [1]. In addition, recurrent VVC can affect nearly 8% of women in the world [2,3]. VVC has been a popular topic, which is a multi-factor infectious disease of women’s lower reproductive tract, mainly caused by *Candida albicans* [4]. Nevertheless, non-*albicans candida* (NAC) species are increasingly isolated worldwide [5]. Among NAC species, *Nakaseomyces glabrata* (former name: *Candida glabrata*), has emerged as an important agent of VVC [6,7], followed by *C. parapsilosis, C. krusei*, and *C. tropicalis* [8,9]. In 2022, the World Health Organization listed *N. glabrata* as one of seven high-priority human fungal pathogens due to its intrinsically reduced susceptibility to fluconazole and other azoles that pose significant public health threats [10].

Molecular typing is important in epidemiological investigation and the prevention of dissemination. Since 2003, multilocus sequence typing (MLST) has been the main method for genotyping and for investigating the epidemiological prevalence of genetic variation in *N. glabrata*, especially when comparability to other studies is beneficial since a curated database exists at www.pubmlst.org/cglabrata [11]. Several studies confirmed the existence of geographically isolated clades when the observed STs were stratified by country of isolation [12,13]. Besides MLST, microsatellite length polymorphisms (MLP) has been considered as a rapid, alternative technique with high discriminatory power (DP) for *N. glabrata* genotyping [12]. MLP is more suitable to differentiate closely related lineages, *e.g. t*o identify clonally in a potential outbreak scenario [11,14].

Despite the global occurrence of *N. glabrata*, the population structure of the aetiological agents associated with VVC is not well investigated. Most studies focusing on genetic diversity and potential association between different *N. glabrata* genotypes and antifungal resistance, or geographical locations have been performed on populations from blood, mouth, anal and faecal swabs, joint fluid, sputum, and other sources [15,16], while vaginal isolates constituted only a few percent (<10% as record in database/www.pubmlst.org/cglabrata) of the strains isolated worldwide.

Suzhou is the most famous historical city, located on the east of China with a subtropical monsoon climate. According to the public security population data in 2023, migrant population from other parts of China accounted for more than 50% in Suzhou, particularly younger generation. The small geographical region, with large migrant population, makes Suzhou an ideal location from which to examine how historical and contemporary factors may influence local population genetic diversity and drug susceptibility of *N. glabrata*.

This study aims to characterize the genetic diversity of *N. glabrata* associated with VVC in Suzhou based on MLST and MLP. In addition, the associations between the most common genotypes and the azole susceptibility are also studied.

## Materials and methods

### Identification of isolates

A total of 204 *N*. *glabrata* isolates were recovered from patients with VVC at the gynaecology clinic of the People’s hospital of Suzhou New District from January to December 2021 as part of a routine hospital procedure. The information for case diagnosis as well as the guidelines based on vaginal samples collected have been described previously [9]. Briefly, cases diagnosed with VVC were based on patients' clinical manifestations, which may include vulvar redness, burning pain, dyspareunia, dysuria, abnormal vaginal discharge, etc., along with a positive fungal examination including hyphae elements or budding cells presented in vaginal secretions by direct microscopy. All samples were collected with sterilized vaginal swabs. Swabs were cultured on Sabouraud glucose agar (SGA; Difco, Detroit, MI, USA) and incubated for 48 h at 35–37°C. The study was conducted in accordance with the Declaration of Helsinki. Ethical approval for the study was obtained from the Ethics Committee of the People’s Hospital of Suzhou New District (The IRB ethics number: 2022023). All participants signed an informed consent to the investigation prior to isolation and use of these samples. All isolates tested in this study were obtained from previous work [9].

All isolates were identified to the species level by sequencing D1/D2 domain of 26S rDNA gene [17], which were deposited in GenBank with accession numbers: OR597664-OR597708, OR597710-OR597783 and OR597785-OR597869.

### MLST and MLP genotyping methods

Total DNA was extracted from pure cultures using CTAB method [17]. Briefly, isolates were resuspended in CTAB buffer supplemented with proteinase K and incubated at 60°C for 1 h to ensure complete lysis. The lysate was then mixed with SEVAG (chloroform:isoamyl alcohol, 24:1) to isolate DNA, followed by precipitation with isopropanol and washing with ethanol. Finally, the purified DNA was stored at − 80°C for preservation.

#### Multilocus sequence typing (MLST)

Six loci (FKS, LEU2, NMT1, TRP1, UGP1, and URA3) were amplified and sequenced as previously described previously [13]. The primers were listed in Table S1. PCRs were performed in 25 µL volumes containing 1 µL of Taq DNA polymerase (Takara Bio Inc.), 2.5 µL of 10× PCR buffer plus 1.5 µL Mg^2+^, 2.5 µL dNTP mixture, 1 µL forward primer and 1 µL reverse primer, 1 µL DNA template. The reaction conditions were as follows: 7 min at 94°C, 30 cycles of 1 min at 94°C, 1 min at the relevant annealing temperature (Table S1), and 1 min at 74°C, followed by 10 min at 74°C. The reactions were performed on an ABI applied biosystems Veriti Dx Thermal Cycler (Thermo Fisher Scientific, USA). The PCR products were sent for sequencing to Bangshi Biopharmaceutical Technology Co., Ltd. (Suzhou, China). Sequences were compared to *N. glabrata* MLST database (https://pubmlst.org/cglabrata/) to assign allele numbers and STs. The newly defined STs refer to the sequence types that were not included in the existing database and numbered by the database manager after depositing the sequences to the MLST database.

Additional MLST typing data stored at the MLST database, including 130 vaginal isolates of *N. glabrata* from China (Shanghai, Hainan, Shenzhen), and 91 vaginal isolates from other regions such as South America, North America, Asia, Europe, and Africa were retrieved from *N. glabrata* MLST database (https://pubmlst.org/cglabrata/) to provide insight into the genetic relationships among the vaginal *N. glabrata* isolates worldwide.

#### Microsatellite analysis (MLP)

Six highly polymorphic microsatellite loci namely RPM2, ERG3, MTI, GLM4, GLM5, and GLM6 were amplified as described previously [14]. The forward primers were labelled with carboxyfluorescein (FAM), hexachlorofluorescein (HEX), and carbosytetramethylrhodamine (TAMRA) (Table S2). PCR was conducted in a 20 μl reaction volume containing 2 μl of DNA template, 1X STR* buffer (Promega, USA), 1.25 U of Ampli Taq gold (Applied Biosystems, France), a reverse primer and a 5′-dye-labelled forward primer at 5 pM of RPM2, GLM4, GLM5, and GLM6, 10 pM for ERG3 and 20 pM of MTI marker. After an initial step of 10 min at 95°C, the PCR included 30 cycles of 95°C for 30 s, 55°C for 30 s and 72°C for 1 min, followed by an additional step of 5 min at 72°C. One microlitre of the PCR mixture was then added to 24 μl of formamide containing 0.5 μl of Genscan LIZ 500 marker (Applied Biosystems, France) and denaturated for 2 min at 95°C. Following the PCR produces was performed by capillary electrophoresis on an ABI3730XL DNA Analyzer (Applied Biosystems). The lengths of the alleles were measured with the GeneMapper Software 5 (Applied Biosystems).

The dendrogram and minimum spanning tree (MSTree) were constructed using BioNumerics 8.0 (Applied Maths). The discriminatory power (DP) was calculated based on Simpson’s index of diversity (http://insilico.ehu. es/mini_tools/discriminatory.power).

### Antifungal susceptibility determination

Antifungal susceptibility was determined on all isolates following the guidelines in CLSI M27-A4 protocol [18]. The antifungals included fluconazole, clotrimazole, voriconazole, and posaconazole (Sigma, Poole, United Kingdom). The concentrations ranged from 0.125 to 64 μg/mL for fluconazole and 0.0313–16 μg/mL for the other three azoles. Briefly, all isolates were cultured on Sabouraud Dextrose Agar (SDA) at 35°C for 24 h, then conidia were harvested with sterile saline, and the final inoculum concentration of the suspension was adjusted to 0.5–2.5 × 10^3^ colony-forming units (CFU)/mL in RPMI 1640 buffered with morpholine propanesulfonic acid. The plates were incubated at 35°C for 24 h and minimum inhibitory concentrations (MIC) were determined. Epidemiological cut-off values (ECVs) were used to classify for triazole susceptibility and to detect non-wild-type isolates. Based on the document M57S-Ed4 [19], epidemiological cut-off values (ECV) 8 μg/mL for fluconazole, 0.25 μg/mL for voriconazole, and 1 μg/mL for posaconazole were considered non-wild type (potentially resistant or less susceptible isolates). There are no CBP or ECVs for clotrimazole in both CLSI and EUCAST guidelines; we refer to MIC > 1 μg/mL for the interpretation of isolates as a non- wild-type [20]. *Candida parapsilosis* ATCC 22,019 and *Candida krusei* ATCC 6258 were used as the quality control.

### Phylogenetic analysis

Clonal complexes (CCs) of 204 isolates of *N. glabrata* were predicted based on MLST data by PHYLOViZ 2.0 software with the global optimal e-BURST (goeBURST) analysis [21] to investigate the evolutionary relationships between isolates. CC is a set of STs that are all believed to be descended from the same founding genotype. STs that could not be assigned to any group were called singletons.

DnaSP v6 was also used to determine the haplotype diversity (*Hd*), nucleotide diversity (π), and possible recombination events based on MLST data [22]. Furthermore, SPLITSTREE v. 4.19.2 [23,24] was used to perform the Pairwise Homoplasy Index (PHI) test on MLST loci data and to build split decomposition trees on concatenated MLST loci data of *N. glabrata*, respectively.

Multilocus linkage disequilibrium (LD) in allelic was assessed by calculating the standardized index of association (*I*^*S*^_*A*_) using Linkage Analysis 3.7 (http://guanine.evolbio.mpg.de/cgi-bin/lian/lian.cgi.pl). LD and linkage equilibrium (LE) would be indicated by a positive value, zero or a negative value, respectively. The population genetic structure was assessed by calculating the variance of pairwise differences (*V*_*D*_) and the 95% critical value (*L*) for *V*_*D*_. If the *V*_*D*_ is less than *L*, the population is in LE, indicating a panmictic population structure; otherwise, the population is clonal population structure and a degree of LD exists [25].

Associations between STs and antifungal susceptibility (WT/non-WT) were evaluated with the chi-square (χ^2^) test and Fisher’s exact test using SPASS Statistics 17.0.

## Results

### MLST and MLP analysis

**Figure 1.**
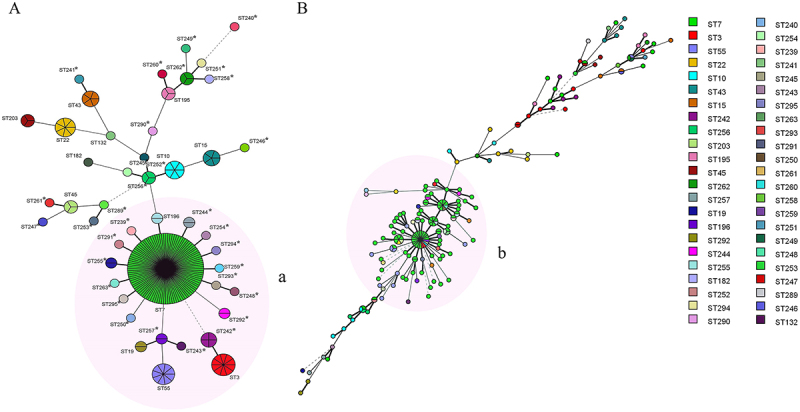
Minimum spanning tree (MStree) analysis based on MLST and MLP genotypes of 204 vaginal *N. glabrata* isolates from Suzhou, China. The group MLP-b consisted of GT51 and its close derivatives majority matched with group MLST-a which consisted of ST7 and its derivatives. A, MStree based on MLST STs data. B, MStree based on MLP GT data. Each circle corresponds to a MLST ST in a and to a MLP GT in B. The colours of circle represent different STs and size of the circles reflects the number of isolates sharing the STs or GTs. The pink area marked as MLST-a and MLP-b, respectively. * referred to the newly defined ST in this study.

A UPGMA tree was generated with MLST data based on genetic distances. A total of 46 STs were identified in 204 isolates of *N. glabrata*, with 32 newly defined STs (ST239-ST263, ST289-ST295) (Figure 1A). ST7 was the most frequently identified, consisting of 51.0% (104/204) isolates, followed by ST3 (10/204, 4.9%) and ST55 (9/204, 4.4%). Notably, 27 STs were unique containing one isolate, of which 25 STs were newly defined in this investigation.

For the 32 newly defined STs, 16 STs contain at least one novel allele, other 16 STs were recombination of six existing alleles. New alleles presented in five loci (except locus NMT1) (Figure S1).

Using microsatellite analyses, the 204 isolates of *N. glabrata* were classified into 146 genotypes (GTs), named GT1-GT146 (Figure 1B, Figure S2, S3). GT51 harboured the largest number of *N. glabrata* isolates (22/204, 10.8%). 127 genotypes were unique GTs (containing only one isolate).

Total 72 MLP GTs shared with MLST ST7 (Figure 1B, Figure S3). The group MLP-b consisted of GT51 and its close derivative majority matched with group MLST-a which consisted of ST7 and its derivatives (Figure 1). The number and proportion of isolates for each genotyping result on 204 isolates of vaginal *N. glabrata* based on MLST and MLP are summarized in Table S3-S4.

The discriminatory power (DP) calculated using Simpson’s index with 204 isolates of vaginal *C. glabrata* reached 0.98 for a combination of 6 MLP markers, and 0.73 for a combination of 6 MLST markers (Figure S4).

### Population genetic analysis

**Figure 2.**
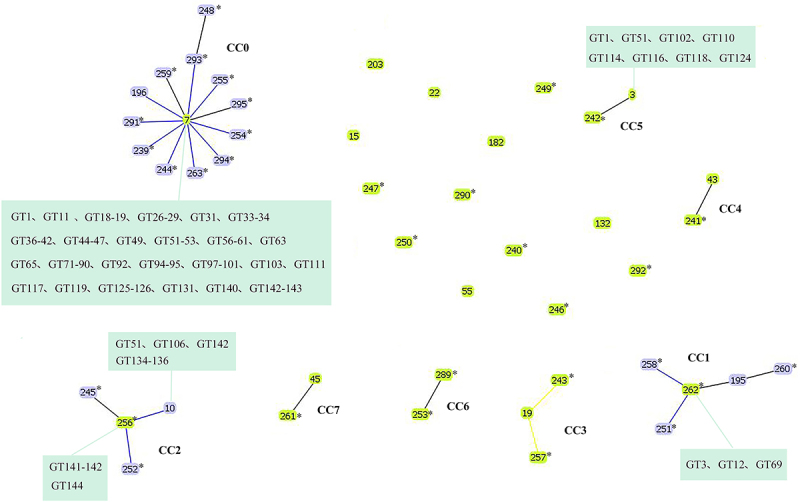
The eBURST analysis based on MLST data of 204 vaginal *N. glabrata* isolates from Suzhou, China. CC0 was the largest CC with ST7 as the primary founder (consisted of 119 isolates, corresponding to 72 MLP-GTs), of which 11 STs were newly defined. The other two STs (ST262 and ST256) were recognized as founders in CC1 and CC2, which both STs were newly defined, suggesting that these isolates have different evolutionary sources than the isolates of CC0. The singletons (7/13 STs) were newly defined STs, suggesting a possible single and recent origin for these isolates in local area. The clonal complexes (CCs) and STs that could not be assigned to any groups were highlighted. The MLP GTs of isolates sharing with the MLST STs were labelled in the box. * referred to the newly defined ST in this study.

eBURST analysis with 204 isolates of vaginal *N. glabrata* based on MLST data resulted in 8 clonal complexes (CCs) and 13 singletons. CC0 was the largest CC with ST7 as the primary founder (consisted of 119 isolates, corresponding to 72 MLP-GTs), of which 11 STs were newly defined (Figure 2). The other two STs (ST262 and ST256) were recognized as founders in CC1 and CC2, which both STs were newly defined, suggesting that these isolates have different evolutionary sources than the isolates of CC0. Strikingly, 53.8% (7/13 STs) the singletons were newly defined STs, suggesting a possible single and recent origin for these isolates locally.

The diversity of the MLST data derived from 204 isolates of *N. glabrata* are shown in Table 1. Haplotype diversity (*Hd*) for the six allele sequences varied between 0.452 and 0.633 and for the concatenated sequences was 0.733; nucleotide diversity (π) for the six alleles and the concatenated sequences were range between 0.00223 and 0.0056, the high haplotype diversity and low nucleotide diversity values indicating a low mutation rate of alleles.

**Table 1.** The diversity of allele sequences in 204 isolates of *N. glabrata* based on MLST data.

Locus	No. of bp Sequenced	No. of haplotype (*Hd*)	No. of variable sites	Nucletide diversity (*π*)	Minimum no. of putative recombination events	P-value (Phi-test)
FKS	589	8 (0.541)	8	0.00274	2	0.06982
LEU2	512	11 (0.561)	12	0.00366	1	0.2286
NMT1	607	10 (0.633)	16	0.00554	4	0.2755
TRP1	419	12 (0.633)	15	0.0056	1	0.6216
UGP1	616	7 (0.452)	9	0.00223	0	1.0
URA3	602	11 (0.597)	12	0.0033	2	0.9748
MLST loci	3345	46 (0.733)	72	0.00375	14	1.578E–9

Note: *Hd: Haplotype diversity*.

Recombination events were estimated with six alleles and the concatenated sequences, which revealed evidence of potential recombination only with the concatenated sequences (PHI: *p* = 1.578 × 10^−9^), suggesting the mixed ancestry of isolates (Table 1).

Linkage disequilibrium analysis of the six alleles showed the positive value of *I*^*S*^_*A*_ (0.2492) and the *V*_*D*_ (2.0530) > *L* (0.9989).

**Figure 3.**
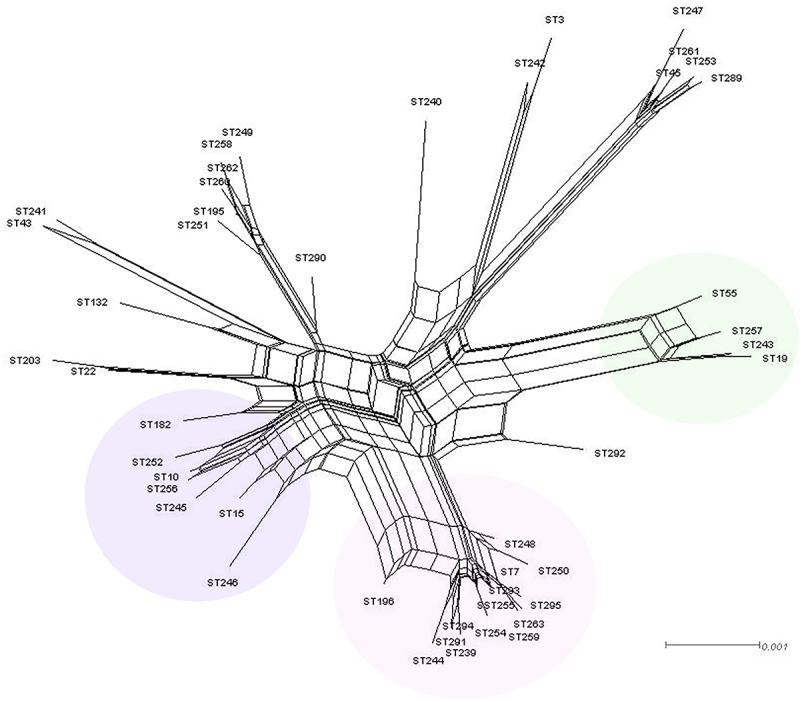
Split decomposition network of 204 vaginal *N. glabrata* isolates from Suzhou, China using MLST data constructed with SPLITSTREE v. 4.19.2. The split decomposition network demonstrated the variability of these isolates with a certain degree of recombination.

The split decomposition network also demonstrated the variability of these isolates with a certain degree of recombination (PHI: *p* = 2.205 × 10^−10^) (Figure 3).

### Comparison of vaginal N. glabrata isolates in China to global isolates

**Figure 4.**
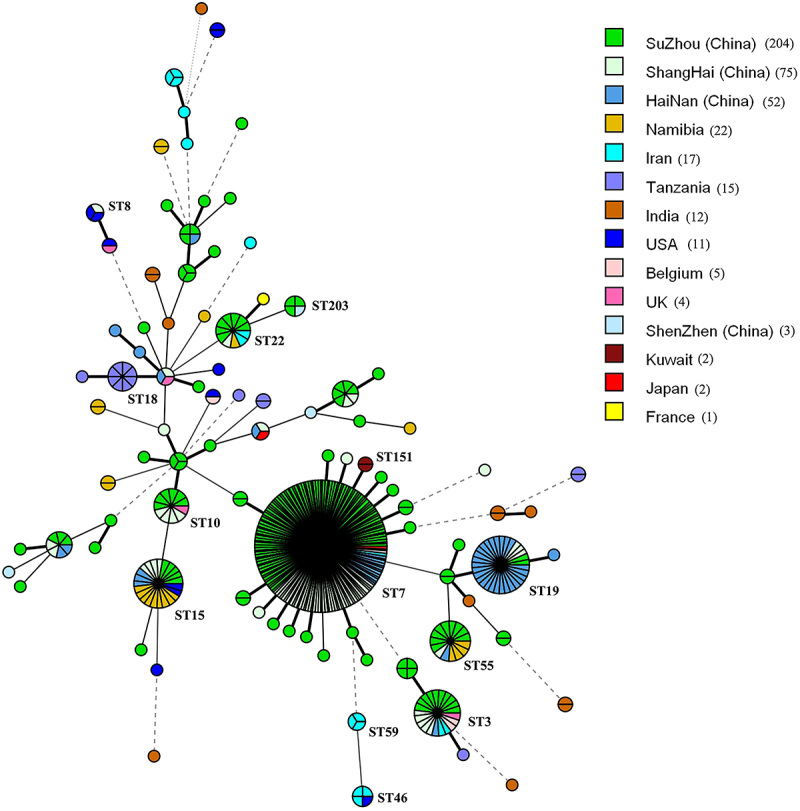
Mstree based on MLST data of vaginal *N. glabrata* isolates from Suzhou, other regions in China, as well as in other countries. Each circle corresponds to a MLST ST and the size of the circles reflects the number of isolates with the STs. The different colours represent different countries.

Combined analysis of the MLST data of the Chinese vaginal isolates (204 isolates from Suzhou, 75 Shanghai, 52 Hainan and 3 Shenzhen) and 91 global isolates from MLST database indicated the vaginal isolates of *N. glabrata* from China were distinct from other countries. Two major independent genetic groups (ST7 and ST19) were recognized among the Chinese isolates (Figure 4). ST7 group was prevalent in China (Suzhou, Shanghai, Hainan), and Asia (such as Japan, Iran), while ST19 was predominant in Hainan in southern China. The genetic profiles of 3 vaginal isolates from Shenzhen, south-east China nested in different branches in the tree. While ST3, ST10, ST15, ST22 presented worldwide (Figure 4).

### Antifungal susceptibility and multilocus sequence typing

Antifungal susceptibility of 204 isolates of *N. glabrata* to 4 azoles summarized in Table 2. Based on the established ECV, 11.3% (23/204) isolates were non-WT to fluconazole, while less than 3% isolates for clotrimazole (1/204), voriconazole (3/204) and posaconazole (5/204) had MIC above the ECV. Six (6/204) isolates were non-WT to more than two azoles.

**Table 2.** Antifungal susceptibility of 204 isolates of vaginal *N. glabrata* to four azoles tested.

Drug	ECV (μg/mL)	MIC range (μg/mL)	MIC_50_\MIC_90_ (μg/mL)	% of non-WT isolates	No. of isolates with MIC of											
					0.0313	0.0625	0.125	0.25	0.5	1	2	4	8	16	32	64
Fluconazole	8	0.25–16	4\8	11.27				1	3	4	66	107	**21**	**2**		
Voriconazole	0.25	0.0313–0.25	0.0625\0.125	1.47	54	123	24	**3**								
Posaconazole	1	0.0313–1	0.25\0.5	2.45	1	5	7	90	96	**5**						
clotrimazole	1	0.0313–1	0.125\0.25	0.49	6	13	83	86	15	**1**						

Then, all isolates were divided into 6 Clusters (C): C1, non-WT phenotype to fluconazole (17 isolates), C2, non-WT phenotype to voriconazole (1 isolates), C3, non-WT phenotype to fluconazole and posaconazole (4 isolates), C4, non-WT phenotype to fluconazole, clotrimazole, and voriconazole (1 isolates), C5, non-WT phenotype to fluconazole, posaconazole and voriconazole (1 isolates), C6, WT phenotype to the 4 azoles tested (180 isolates).

**Figure 5.**
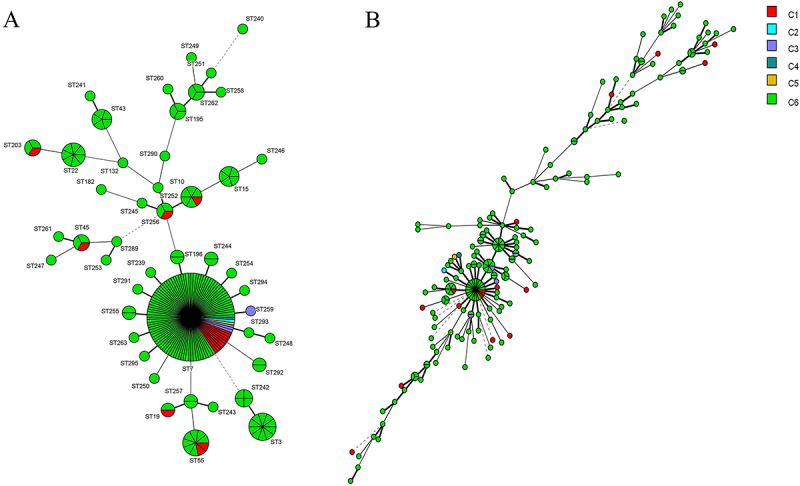
Mstree conducted by 204 vaginal *N. glabrata* isolates from Suzhou, China showing the relationship between the MLST ST (A)/MLP GTs (B) and susceptibility patterns of four azoles tested. Each circle corresponds to a MLST ST in a and a MLP GT in B. The colour represented different groups as follows: C1, non-WT phenotype to fluconazole (17 isolates), C2, non-WT phenotype to voriconazole (1 isolates), C3, non-WT phenotype to fluconazole and posaconazole (4 isolates), C4, non-WT phenotype to fluconazole, clotrimazole and voriconazole (1 isolates), C5, non-WT phenotype to fluconazole, posaconazole and voriconazole (1 isolates), C6, WT phenotype to the 4 azoles tested (180 isolates). The circle size corresponds to the isolate number. Dark, dashed, and thin lines surrounding ST circles represent the genetic distance.

Distribution of genotyping (both MLST-STs and MLP-GTs) and antifungal susceptibility phenotype to 4 azoles tested were shown in the MStree (Figure 5 A, B). The predominant ST7 presented in all non-WT phenotype clusters (C1–C5) that 10 isolates were assigned in C1, 6 in C2-C5; however, another 7 isolates in C1 harboured 6 different STs rather than ST7 (Figure 5A). ST7 was the dominant sequence type in *C. glabrata* isolates with cross-resistance to azoles. However, no association between STs/GTs and non-WT phenotypes to azoles was detected by chi-square (χ^2^) test and Fisher’s exact test (*p* > 0.05).

## Discussion

Since *C. glabrata* shares a recent common ancestor with several Saccharomyces species and belongs to a clade different from that of other Candida species, the new taxonomical classification in 2022 changed its name as *Nakaseomyces glabrata* belonged to the *Nakaseomyces*, *Saccharomycetes*, *Ascomycota* [26]. Understanding the local population structure and microevolution of vaginal *N. glabrata* is important to investigate the ecological patterns of VVC spreading. The main objective of the present study was to analyse the population genetic diversity of *N. glabrata* isolated from VVC patients in Suzhou, in eastern China.

Here, the MLST analysis of 204 vaginal isolates of *N. glabrata* revealed that ST7 was the predominant ST along with a considerably high proportion (32/46, 69.6%) of novel/unknown STs when compared with the existing MLST database which has not been found anywhere else worldwide except Suzhou, reflecting a relatively high level of genetic variation of vaginal *N. glabrata* isolates in Suzhou. ST7 has been reported as prevalence ST in *N. glabrata* with systemic infection in Asia, including China [12,27]. Prevalence of ST7 in vaginal *N. glabrata* population in China demonstrated that endemicity of strains with particular traits is maintained due to selective pressure within the area, highlighting the significance of acquiring local data.

Certain STs may be associated with specific body sites. For instance, Odds et al. [28] reported a correlation between genotypes and isolation at particular body sites for *C. albicans* that showed significant differences in the proportions of isolates from blood, commensal carriage, and superficial infections.

Enache-Angoulvant A et al. [29] analysed the genetic relationships between bloodstream and digestive tract *N. glabrata* isolates by multilocus microsatellite typing on a panel of 136 blood isolates and 34 digestive tract isolates from patients, it was shown that digestive tract isolates differed from blood culture isolates by exhibiting a higher genotypic diversity associated with different allelic frequencies. Previous work has reported that vaginal *N. glabrata* strains showed a greater genetic diversity than that of non-vaginal strains assessed using MLST genotyping [27], as the commonly accepted that *N. glabrata* can be a persistent commensal of the human vaginal tract carriage [30], our data demonstrated that the genetic evolution of vaginal *N. glabrata* isolates may be different from that of from systemic infection by exhibiting a higher genetic diversity.

In our study, a higher proportions of vaginal tract isolates corresponding to unique STs (27 STs among the unrelated 46 STs, 58.7%), in particular, the new unique STs (25 new unique STs in 27 unique STs) in Suzhou raise the question of their propensity for epidemiologic spread and capacity to adapt to the other conditions such as systematic (for example bloodstream) or digestive tract. Further basic work to correlate these observations with virulence is warranted. Contrasting STs distribution patterns were observed between the Chinese and non-Chinese vaginal isolates (Figure 4). This could be due to the limited vaginal *N. glabrata* isolates available in the existing MLST database, as well as different environmental and demographic factors prevailing in the region. The geographic origin of isolates belonging to ST7 and ST19 appeared to be distributed mainly in Asia (e.g, Suzhou, Shanghai, and Hainan in China).

Microsatellite genotyping of *N. glabrata* showed a similar picture: the majority of isolates that formed the largest cluster defined in the MLST MStree also formed similar clusters in the MLP MStree (Figure 1, MLP-b vs MLST-a). However, the MLST method did not, for the most parts, discriminate between isolates in a group (Figure 1). MLP had a higher discriminatory power (0.98) than MLST (0.73) in the molecular typing of *N. glabrata* (Figure S4), and both were higher than that from a previous study [12]. Nevertheless, our results showed a generally good congruence between MLST and microsatellite genotyping for *N. glabrata*, which is in agreement with previous studies [11,12].

Based on eBURST, ST7 could be the founder of other STs linked to (Figure 2). This cluster consisted of 58.3% (119/204) isolates studied and displays the likely pattern of recent evolutionary descent of 12 STs within the CC0, suggesting a gradual diversification of ST7 by mutation and recombination. Furthermore, split decomposition analysis of *N. glabrata* on MLST loci revealed evidence of recombination, suggesting that cryptic sexual reproduction may be present (Figure 3, Table 1). Evidence of potential recombination events might also contribute to high intra-species genetic variability among isolates.

Suzhou has been going through rapid development in the past 20 years, with many young workers coming from different regions of the country. According to national population data in 2023, 50% of Suzhou population is non-native people, particularly younger generation (e.g. around 25 in age). Thus, travel and human migration events could potentially facilitate the introduction of *N. glabrata* from different regions to Suzhou. Our data suggested that the genetic evolution of vaginal *N. glabrata* isolates in Suzhou may be different in terms of diversity due to its specific conditions such as tourism, labour migration and ecological isolation. Our results were in agreement with a recent published article by Wang et al. This study investigated a large population structure of *N. glabrata* through genome sequencing of 80 clinical isolates obtained from six tertiary hospitals in Qatar and compared to global samples showed that the Qatari *N. glabrata* population was consistent with the significant role of recent anthropogenic activities in shaping its population structure [31].

Our results showed that only 15.7% of vaginal *N. glabrata* isolates were non-WT to four azoles tested, which was consistent with those reported from Kiasat N (2019) [32], Chen (2022) [27] and Delliere et al. (2016) [33]. Based on CBP of *N. glabrata* to fluconazole (MIC < 64ug/ml), no isolate was fluconazole resistant in our study. Kiasat N et al. reported a similar result that only one isolate (in total 61 isolates of vaginal *N. glabrata*) was fluconazole resistant using EUCAST methods [32]. A study collected in total > 480 isolates of *N. glabrata* from candidemia patients among 77 hospitals in China over a period of 3 years has shown an overall fluconazole resistance rate of 10.2% (3.1–11.4%) and a decrease in voriconazole susceptibility in *N. glabrata* [34]. However, a study from Italy reported that 30% resistant rate of fluconazole to *N. glabrata* isolated from VVC [35].

The majority ( > 95%) of these isolates remained susceptible to clotrimazole (MIC < 1 μg/mL) in our study which is similar to those reported by Ziba Abbasi Nejat et al. that 1/21 isolates of vaginal *N.glabrata* had MIC > 4 ug/ml for clotrimazole by CLSI methods [36]. While a recent study by Maqsood Ali reported that 20.8% resistant rate for vaginal *N. glabrata* to clotrimazole using the disk diffusion test of the Kirby – Bauer technique [37]. Thus, it underscores the importance of regular susceptibility testing for vaginal *Candida* isolates. Of note, the proportion of isolates (26.1%, 6/23) that non-WT to fluconazole cross-resistance to other azoles was high. VVC is treated with topical clotrimazole, imidazoles, and polyenes in the form of pessaries, or creams, as well as oral triazoles [4]. However, the misuse or overuse of antibiotics for patients without prescription in such prophylactic, empirical, and pre-emptive therapies could lead to long-term exposure to antifungal drugs, potentially resulting in the emergence of multidrug-resistant fungi [38].

Although there was no association between STs and susceptibility to the azoles tested in this study, it was noticed that the majority of vaginal *N. glabrata* isolates with non-WT azoles phenotype (G1–G5) belonged to ST7 (Figure 5), which is consistent with those reported from previous studies [27,39]. Whereas it was conflicting with the study by Kiasat N et al. [32], which demonstrated a positive association between *N. glabrata* populations and the predominant genotype (GT27) with resistance phenotype to azoles. This discrepancy may be due to different methodologies in genotyping assays (microsatellite analysis) or origins and sources of clinical isolates. Further study with additional isolates is needed to investigate the discrepancy from this observation.

One of the limitations in this study was that this was a single centre, further studies with additional samples from different hospitals are required to investigate comprehensively the genetic diversity of the vaginal isolates in Suzhou area, China. Moreover, the number of vaginal *N. glabrata* isolates in the existing MLST database is limited and fragmented, plus impacting the investigation on the geographic distribution of vaginal *N. glabrata* isolates globally.

## Conclusions

Our data provide new insights into the variability and evolution of *N. glabrata* associated the local Chinese populations. The relatively high genetic diversity among vaginal population of *N. glabrata* in Suzhou may be related to high dynamism of the *N. glabrata* genome with recombination, and rapid adaptation to host and geographical selection. MLST and MLP are two effective typing methods to illustrate the genetic diversity of vaginal *N. glabrata* isolates. Routine antifungal susceptibility testing for vaginal *Candida* isolates would lay a crucial role in the management of VVC.

## Supplementary Material

Figure S4.jpg

Figure S3.jpg

Figure S2.jpg

Figure S1.jpg

## Data Availability

Raw sequence data (D1/D2 gene sequences) are available on the NCBI website under GenBank accession number from OR597664-OR597708, OR597710-OR597783 and OR597785-OR597869. All raw data in this study are available in figshare under doi number: 10.6084/m9.figshare.28578770 (https://doi.org/10.6084/m9.figshare.28578770.v1). The public data included in this study were sourced from the PubMLST.org website (www.pubmlst.org/cglabrata), they are openly available for unrestricted reuse.
