# Supplementary figures and images for: Molecular epidemiology of Nakaseomyces glabrata associated with vulvovaginal candidiasis revealed high genetic variability and the presence of novel genotypes in China
Source: Virulence. 2025 Aug 3;16(1):2543058. doi: 10.1080/21505594.2025.2543058 (PMC12330260; doi:10.1080/21505594.2025.2543058)

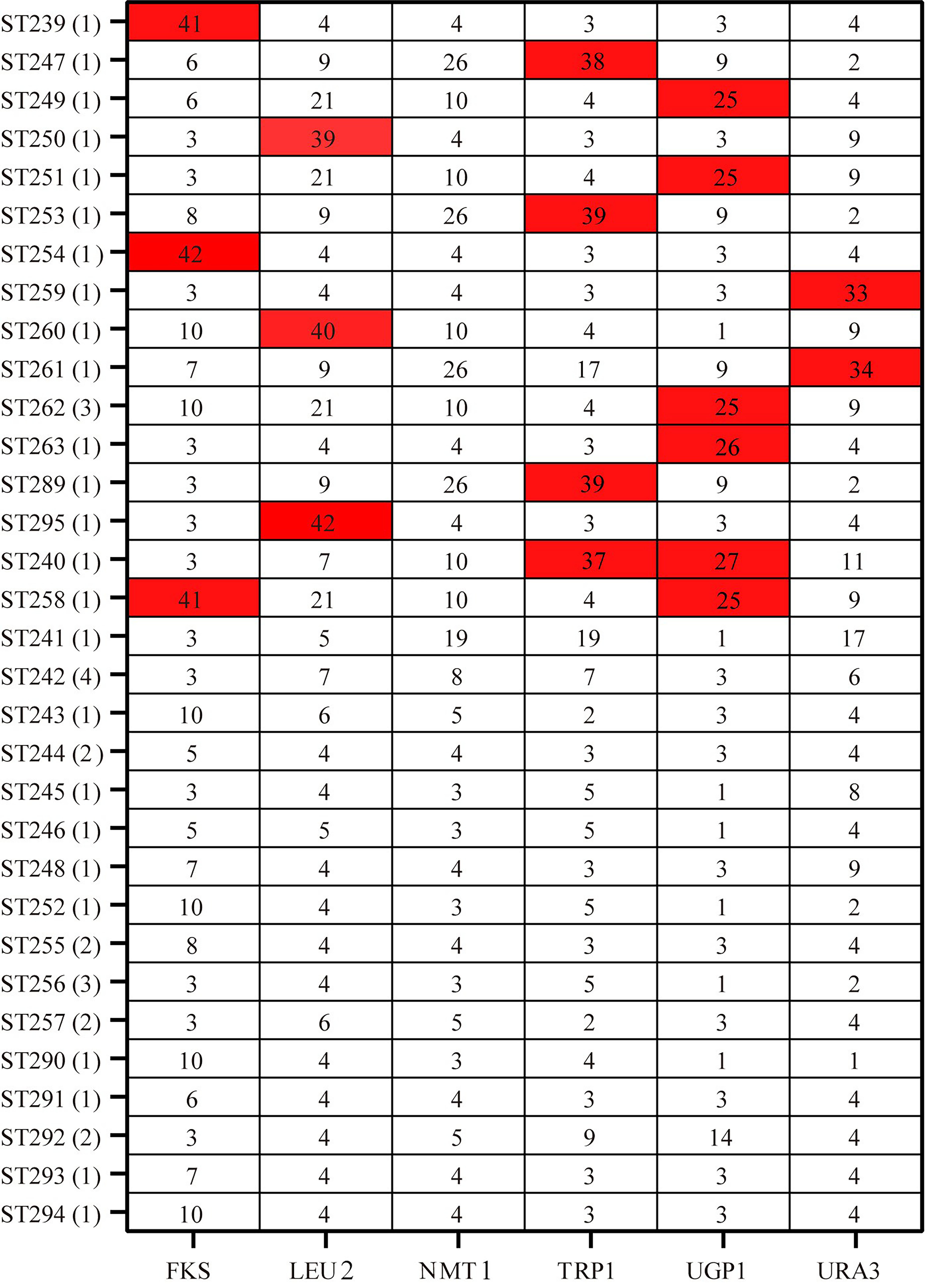

Supplement: Figure S1.jpg [file KVIR_A_2543058_SM3894.jpg]
